# Differences in PfEMP1s recognized by antibodies from patients with uncomplicated or severe malaria

**DOI:** 10.1186/s12936-016-1296-4

**Published:** 2016-05-05

**Authors:** Michael F. Duffy, Rintis Noviyanti, Takafumi Tsuboi, Zhi-Ping Feng, Leily Trianty, Boni F. Sebayang, Eizo Takashima, Fransisca Sumardy, Daniel A. Lampah, Louise Turner, Thomas Lavstsen, Freya J. I. Fowkes, Peter Siba, Stephen J. Rogerson, Thor G. Theander, Jutta Marfurt, Ric N. Price, Nicholas M. Anstey, Graham V. Brown, Anthony T. Papenfuss

**Affiliations:** Department of Medicine, Royal Melbourne Hospital, The University of Melbourne, The Peter Doherty Institute for Infection and Immunity, Melbourne, Victoria Australia; The Eijkman Institute for Molecular Biology, Jakarta, Indonesia; Division of Malaria Research, Proteo-Science Center, Ehime University, Matsuyama, Ehime Japan; Bioinformatics Division, The Walter & Eliza Hall Institute of Medical Research, Parkville, Victoria, Australia; Department of Medical Biology, University of Melbourne, Parkville, Victoria Australia; Timika Malaria Research Program, Papuan Health and Community Development Foundation, Timika, Papua Indonesia; Centre for Medical Parasitology, University of Copenhagen, Copenhagen, Denmark; The Burnet Institute, Melbourne, Victoria Australia; The Papua New Guinea Institute for Medical Research, Madang, Papua New Guinea; Global and Tropical Health Division, Menzies School of Health Research, Charles Darwin University, Darwin, NT Australia; Centre for Tropical Medicine and Global Health, Nuffield Department of Clinical Medicine, University of Oxford, Oxford, UK; The Nossal Institute for Global Health, The University of Melbourne, Parkville, Victoria Australia; Peter MacCallum Cancer Centre, East Melbourne, Victoria, Australia; Sir Peter MacCallum Department of Oncology, University of Melbourne, Melbourne, Victoria Australia

**Keywords:** Severe malaria, *var* genes, PfEMP1

## Abstract

**Background:**

*Plasmodium falciparum* erythrocyte membrane protein 1 (PfEMP1) variants are encoded by *var* genes and mediate pathogenic cytoadhesion and antigenic variation in malaria. PfEMP1s can be broadly divided into three principal groups (A, B and C) and they contain conserved arrangements of functional domains called domain cassettes. Despite their tremendous diversity there is compelling evidence that a restricted subset of PfEMP1s is expressed in severe disease. In this study antibodies from patients with severe and uncomplicated malaria were compared for differences in reactivity with a range of PfEMP1s to determine whether antibodies to particular PfEMP1 domains were associated with severe or uncomplicated malaria.

**Methods:**

Parts of expressed *var* genes in a severe malaria patient were identified by RNAseq and several of these partial PfEMP1 domains were expressed together with others from laboratory isolates. Antibodies from Papuan patients to these parts of multiple PfEMP1 proteins were measured.

**Results:**

Patients with uncomplicated malaria were more likely to have antibodies that recognized PfEMP1 of Group C type and recognized a broader repertoire of group A and B PfEMP1s than patients with severe malaria.

**Conclusion:**

These data suggest that exposure to a broad range of group A and B PfEMP1s is associated with protection from severe disease in Papua, Indonesia.

**Electronic supplementary material:**

The online version of this article (doi:10.1186/s12936-016-1296-4) contains supplementary material, which is available to authorized users.

## Background

PfEMP1 is the immunodominant antigen of the malaria parasite *Plasmodium falciparum* expressed on the surface of the infected erythrocyte (IE). Adherence of this molecule to host receptors expressed on endothelial cells, uninfected erythrocytes and placental syncytiotrophoblasts facilitates sequestration of IE in vascular tissues, avoiding destruction in the spleen [1–3]. PfEMP1 molecules are encoded by the *var* multigene family [1–3]. Individual parasites have approximately 60 *var* gene variants and switching between single, transcribed *var* genes leads to changes in cytoadhesive phenotype as well as clonal antigenic variation and immune escape. *var* gene repertoires differ among isolates [4] and immunity to malaria is dependent on acquisition of antibodies to a range of PfEMP1 variants [5–8]. Immunity to both cerebral malaria [9] and non-cerebral, severe malaria [10] is acquired much more rapidly than immunity to uncomplicated malaria. Parasites that cause severe disease appear to express a conserved subset of variant antigens that are encountered earlier in life and that are thus more widely recognized by sera from semi-immune children than parasites causing uncomplicated disease [11, 12].

PfEMP1s contain combinations of Duffy binding-like domains (DBLα, β, γ, δ, ε, ζ and x) and cysteine rich inter-domain regions (CIDRα, β, γ and δ) [13]. Some DBL and CIDR domain subtypes mediate adhesion to different host receptors (reviewed in [14, 15]), and some are organized in semi-conserved domain cassettes (DC) that are present in most parasites [4]. *var* genes are also classified using their upstream sequence into groups A, B, C [16, 17] which comprise 20, 60 and 20 % respectively of the *var* gene repertoire [4]; the unique *var* gene called *var2csa* has a different upstream sequence (ups E) and is only involved in malaria during pregnancy [18].

The expression of particular subtypes of DBLα domains in severe malaria suggests severe disease may be preferentially caused by a restricted subset of *var* genes [19, 20]. Increased expression of group A and B *var* genes has been associated with clinical, but not specifically severe malaria in Papua New Guinea (PNG) [21, 22] and with severe malaria in Africa [23]. Cerebral malaria in Africa was associated with increased expression of group A [20, 24, 25] or group B [26] *var* genes.

Consistent with its having a role in severe malaria, PfEMP1s encoded by group A and B *var* genes appear to be widely expressed by parasites that infect non- or semi-immune individuals. Antibodies from older children preferentially recognized PfEMP1s encoded by Group A *var* genes, indicating previous exposure [27]. Group A and B *var* genes dominated infection of a naive individual [28], and more individuals develop antibodies to group A PfEMP1s than group B or C, and do so at a younger age [29].

Group A and B *var* genes also encode adhesion phenotypes associated with severe disease. In Africa the adhesion phenotype of rosetting is associated with severe malaria [14] and increased expression of group A *var* genes [19, 21, 25]. Some group A and B PfEMP1s can bind to intercellular adhesion molecule 1 (ICAM-1) [30, 31], and ICAM-1 expression was up-regulated in brain endothelium and co-localized with sequestered IEs in cerebral malaria patients [32]. IE adhesion to ICAM-1 has variously been associated with cerebral malaria [33], clinical but not severe malaria [34] or inversely correlated with severe disease [35].

Another phenotype associated with severe disease is adhesion to endothelial protein C receptor, EPCR [36, 37]. Parasite isolates from African children with severe malaria bound EPCR and expressed DC8 or DC13 *var* genes [36, 38]. DC8 and DC13 PfEMP1s are primarily group B and A, respectively [4], and contain members of the subset of CIDRα1 domain types, which bind EPCR [36, 37]. Sera from African children with uncomplicated malaria recognize PfEMP1s containing DC8 and DC13 at higher levels than PfEMP1s without DC8 or 13, but it is unclear whether severe malaria specifically induces antibodies to DC8 and DC13 [39, 40].

DC5 PfEMP1s, which are nearly all group A, are recognized by sera from semi-immune children in a similar manner to other severe malaria associated isolates [27], and DC5 expression increased markedly during infection of a naive volunteer [41]. African children acquired antibodies to DC5 more rapidly than to other PfEMP1 domains which is consistent with widespread expression of DC5 in non-immune individuals [29]. Antibodies reactive with DC5 also correlated with protection from malaria episodes [42]; however, the evidence directly linking DC5 PfEMP1 expression and adhesion phenotype to severe malaria is less clear. High levels of DC5 sequence expression have been detected in severe malaria, but only together with expression of either DC8 or 13 sequences [38] and it is conceivable that these DC5 and DC8 or DC13 sequences were present on the same *var* genes. DC5 PfEMP1s bind platelet-endothelial cell adhesion molecule 1 (PECAM1) [43] and IE adhesion to PECAM1 has been implicated in cerebral malaria [44, 45]. However, IE adhesion to PECAM1 is also commonly found in samples from patients with uncomplicated malaria [46] and DC5 PfEMP1s were not expressed by parasites selected for adhesion to brain endothelium [39, 40]. A minority of *var* genes containing DC5 do encode EPCR-binding CIDRα1.5 domains although these domains are not part of the DC5 cassette [4].

Thus several promising candidates have emerged as members of the restricted population of PfEMP1s responsible for severe malaria, but the relative contributions of group A and B, and of DCs 8, 13 and 5 remain unclear. In particular very little is known about PfEMP1s in severe disease in the Asia Pacific region. Determining whether conserved PfEMP1sequences elicit protection from severe malaria disease globally is a priority for vaccine research. In this study plasmas from Papuan patients with severe or uncomplicated malaria were analysed for their reactivity with PfEMP1 polypeptides representative of the different groups and DCs including several expressed by parasites causing cerebral malaria.

## Methods

### Patient samples

Two ml of venous blood was collected from a 5-year old female patient with cerebral malaria in PNG. The IEs were separated from white blood cells by Plasmodipur column filtration (Europroxima) as per the manufacturer’s instructions. The IEs were lysed in TRIzol^**®**^ (Life Technologies), incubated at 37 °C for 5 min and then stored at −80 °C. Venous samples were collected from patients with severe (n = 28) and uncomplicated (n = 35) malaria attending a healthcare facility in Timika, Papua Province, Indonesia. Plasma was separated from blood by centrifugation and stored at −20 °C. This area has unstable malaria transmission with estimated annual parasite incidence of 450 per 1000 population and symptomatic illness in all ages [47]. Severe malaria was defined as peripheral parasitaemia with at least one modified World Health Organization (WHO) criterion of severity [48]. Twenty-six of the 28 patients with severe malaria had parasitemias greater than 1000/μL, which is a previously-derived threshold that predicts clinical disease in northern Papua [49]. Thus incidental parasitemia is unlikely in these 26 severe malaria patients but cannot be excluded in the two severe malaria patients with parasitemias less than 1000/μl.

### Ethics

Informed consent was provided by all participants. The study was approved in Indonesia by the Eijkman Institute Research Ethics Commission (project number 46), in Australia by the Melbourne Health Human Research Ethics Committee (project number 2010.284) and Human Research Ethics Committee of the NT Department of Health & Families and Menzies School of Health Research, Darwin, Australia (HREC 2010–1396), and in PNG by the Government of PNG Medical Research Advisory Committee (MRAC no. 11.12).

### RNA extraction and RNAseq

Erythrocytes in TRIzol^**®**^ were thawed at 37 °C, chloroform (1/5th of the TRIzol^**®**^ volume) was added and vortexed 15 s, the solution was then subjected to centrifugation at 12,000×g for 30 min at 4 °C and the aqueous supernatant was aspirated and mixed with an equal volume of 70 % ethanol in RNase free water. This solution was then directly applied to RNeasy mini columns (QIAGEN), and RNA purification was performed with on-column DNasing as per the manufacturer’s instructions. Eluted RNA was oligo dT purified and used to generate a 65 bp paired end RNAseq library using the standard Illumina protocol. The library was sequenced on an Illumina GAII at Geneworks (Adelaide).

Alignments were performed using Subreads [50]. Reads were first aligned to the 399 full length *var* genes that were previously described in seven *P. falciparum* isolates [4] and mapped reads were extracted. Reads were also aligned to the 3D7 *P. falciparum* genome and unmapped reads were extracted to include any *var* that may have been missed. The reads that mapped to *var* genes and the reads that did not map to the 3D7 genome were then merged, digitally normalized using khmer [51], and assembled using Oases [52]. Assembled transcripts were subjected to a second round of *de novo* assembly using Cap3 [53] to assemble contigs and extract consensus sequences from the contigs. Contigs were aligned to the domains from the 399 *var* genes with BLAST (version 2.2.25). The transcript abundance was determined by aligning the reads again to the assembled transcripts.

### Protein expression

Proteins for Luminex assays were expressed in baculovirus-transfected insect cells as previously described [54]. Proteins analysed by Luminex were IT4var02 protein 1 DBLγ12DBLδ5CIDRβ3DBLβ9, PF11_0008 DBLδ5CIDRβ4, IT4var02 protein 2 DBLδ5CIDRβ3, HB3var05 DC16 DBLα1.6CIDRδ1, PFI1820w var3 DBLα1.3 DBLε8, PFD0020c DBLβ12 DBLγ6, IT4var20 DBLα2CIDRα1.1DBLβ12DBLγ6DBLδ1CIDRβ1, PF11_0007 DBLα0.15CIDRα3.2DBLδ1CIDRβ1, PFL0020w DBLγ14DBLζ5DBLε4, IT4var13 DC 9 DBLα0.3CIDRα5DBLβ5DBLδ9CIDRγ9DBLγ11DBLζ4.

PfEMP1 domains identified by RNAseq as expressed by the parasites infecting the PNG CM patient were amplified from gDNA purified from the patient’s blood for use as ELISA antigens. The sequences were then cloned into the plasmid pEU-E01H-N1 (encoding an N terminal His-tagged protein; CellFree Sciences Matsuyama, Japan) [55] and expressed in the wheat germ cell-free expression system (CellFree Sciences) and purified on Ni-nitrilotriacetic acid agarose columns (Qiagen, Valencia, CA) as previously described [55, 56].

Antigens used for the ELISA included proteins one to seven that were encoded by *var* gene sequences transcribed by parasites infecting the cerebral malaria patient from PNG. Proteins 1 and 3 were encoded by 352 bp and 123 bp fragments of a contig with homology to the DC5 DBLβ7_D8 of Dd2var4 (ranked 33rd by transcript abundance) (Table 1). Protein 2 was encoded by a 861 bp orthologue of DC5 DBLδ5_D6_Dd2var4 that incorporated two contigs that were ranked 4th and 9th by abundance (Table 1). Protein 4 was encoded by a 697 bp fragment of a contig with homology to a DC8 DBLγ6_D3_Dd2var47 (ranked 5th by abundance). Protein 5 was encoded by a 186 bp fragment with homology to DBLγ10 of raj116 and was ranked 34th by abundance. Protein 6 was encoded by a 425 bp contig that incorporated two non-overlapping contigs with homology to NTSB3_DBLα0.11 of igh_var31 and DBLα0.15 of raj116_var34 (ranked respectively14th and 21st by abundance). Protein 7 was a 142 bp cloned orthologue of DBLδ1**_**D4_PFCLIN_var24 (ranked 96th by abundance).

**Table 1:** *var* sequences transcribed by parasites infecting a Papua New Guinean cerebral malaria patient

*var* Homologs^a, b^	Notes	RPK^c^	Contig bp	Homology bp	E value	% Identity
CIDRα2.4_D3_MAL7P1.55		7384	425	131	5.44E−34	84
DBLδ1_D4_ighvar31_CIDRγ9_D5_ighvar35		5174	1553	133, 410	2.52E−32, 1E−100	82.7, 79
DBLδ9_D5_PFCLINvar74		4369	583	303	1.05E−44	76.2
*DBLδ5_D6_Dd2var4*	DC5 ELISA protein 2	4227	273	219	1.19E−52	80.4
*DBLγ6_D3_DD2var47–DBLγ2_D5_DD2var42*	DC8 ELISA protein 4	4130	1807	190, 488	2.57E−58, 6.86E−148	86.3, 85
DBLβ6_D5_igh_var27		3562	395	258	3.17E−68	81.8
DBLε1_D6_PFCLINvar76	var1csa	3340	1025	429	0	100
CIDRα3.1_D3_DD2var50		2973	550	86.1	3.24E−57	86.1
*DBLδ5_D6_Dd2var4*	DC5 ELISA protein 2	2891	156	123	7.29E−46	92.7
CIDRβ1_D8_HB3var1		2814	650	161	7.44E−41	82.6
CIDRα3.1 pf08–0106–DBLδ5_D5_igh_var30		2302	883	167, 310	1.51E−38, 7.32E−68	85.6, 80.6
DBLγ15_D5_PFCLINvar76 (var1csa)		2199	246	230	2.56E−117	100
DBLγ17_D4_DD2var43–DBLδ5_D5_HB3var2	DC5	2153	626	192, 250	4.51E−56, 4.23E−31	87.5, 73.6
*NTSB3_D1_igh_var31*–*DBLα0.11_D2_AAB60251*	ELISA protein 6	2085	189	93, 80	1.03E−19, 1.03E−19	81.7, 85
DBLγ10_D5_HB3var34	DC17,21,22	1994	344	193	2.94E−74	91.7
CIDRβ1_D7_raj116_var11	DC8	1980	606	408	1.98E−60	73.8
DBLβ1_D4_igh_var19	DC8	1935	402	344	1.29E−98	83.7
DBLδ1_D4_HB3var50		1911	302	229	3.32E−60	82.1
DBLδ1_D4_IT4var39		1894	216	203	2.31E−41	76.4
DBLβ6_D4_igh_var9		1883	437	251	3.1E−75	85.3
*DBLα0.15_D2_raj116_var34*	ELISA protein 6	1871	132	125	6.84E−39	87.2
DBLδ1_D4_IT4var46–DBLγ4_D5_raj116_var11		1867	1461	115, 258	4.57E−35, 3.5E−106	87.8, 84.1
DBLα0.1_D2_HB3var30		1846	338	215	5.37E−43	82.8
DBLγ6_D5_IT4var32b	DC8	1833	307	178	1.45E−49	83.7
DBLγ13_D5_HB3var21		1822	828	402	1.09E−114	90.7
CIDRβ4_dd2var22–DBLβ3_D4_HB3var3	DC5-multi domain contig	1819	1152	707, 339	3.35E−86, 1.34E−59	70.9, 75.2
CIDRα2.9_D3_raj116_var14 –DBLδ1_D6_IT4var32b		1741	534	43, 196	1.83E−15, 9.49E−32	100, 76
DBLα0.19_D2_Itvar66-CIDRα2.4_D3_raj116_var29		1729	410	96, 279	3.33E−36, 2.73E−56	93.8, 79.2
DBLδ1_D4_igh_var20		1724	480	237	5.43E−57	79.7
DBLδ1_D4_IT4var47		1714	263	172	3.28E−46	83.7
DBLδ1_D4_igh_var18		1696	289	227	2.78E−48	82.8
DBLδ1_D4_PFD1005c		1649	191	191	5.39E−36	76.9
*DBLβ7_D8_Dd2var4*	DC5 ELISA proteins 1 & 3	1644	859	793	1.46E−152	78.6
*DBLγ10_D4_PFCLINvar71*	ELISA protein 5	1587	511	204	2.3E−71	89.2
DBLβ5_D4_IT4var16-DBLδ1_D4_raj116_var32		1579	447	135, 62	5.743E−53, 1.11E−17	93.3, 90.3
DBLδ1_D4_raj116_var29		1573	309	297	2.3E−43	73.4
DBLβ12_D4_raj116_var11–DBLγ11_D4_raj116_var17		1534	712	215, 178	7.05E−47, 1.45E−36	79.5, 80.3
DBLβ11_D4_IT4var35–DBLγ3_D7_HB3var4		1520	931	163, 131	3.27E−65, 3.5E−33	93.3, 83.2
DBLε3_D8_raj116_var29		1508	179	60	3.63E−25	100
DBLδ1_D7_IT4var22		1454	388	351	6.02E−52	74.1
DBLδ1_D5_igh_var5		1438	281	287	1.07E−40	70.5
DBLβ3_D4_AAQ73927–DBLγ4_D5_raj116_var8		1428	458	171, 184	8.18E−45, 2.2E−39	83, 79.9
DBLε10_D8_IT4var4		1392	442	138	3.35E−43	87.7
DBLδ1_D4_PFCLINvar28 (var2csa)		1378	506	138	9.7E−51	91.3
DBLγ8_D7_PFCLINvar76 (var1csa)		1377	244	200	1.05E−71	92
DBLγ11_D5_DD2var52–DBLδ1_D4_PFCLINvar36		1351	910	322, 84	3.21E−47, 1.12E−27	73.6, 91.7
DBLα1.6_D2_DD2var22		1320	111	102	1.7E−26	84.3
DBLα0.15_D2_HB3var18		1298	352	306	1.28E−53	77.8
CIDRβ1_D8_IT4var22		1297	431	224	9.97E−69	89.7
CIDRα1.5_D3_ighvar30–DBLβ7_D7_PFCLINvar69	EPCR binding	1283	2183	(31, 42, 66), 681	(1.06E−3, 3.7E−3, 1.21E−15), 1.33E−110	(87.1, 90.5, 98.5), 73.6

Shown are the 50 highest-ranked transcripts

^a^The domains that were expressed for analysis by ELISA are in italics

^b^Domain annotation is as per [4]: domain subtype_domain (D) position within the PfEMP1 numbered from the most N terminal DBL/CIDR domain_*P. falciparum* isolate name *var* gene name

^c^The transcripts are ordered by coverage [reads mapped per kb assembled transcript (rpk)]

Proteins 1, 2 and 3 were most closely related to the DC5 domain *var* gene Dd2var4. This gene has group A-like coding features, e.g. head structure and ATS, and is the only DC5 with a group B-like upstream sequence, the other 11 all being group A [4]. Therefore, proteins 1, 2 and 3 were classified as group A for all subsequent analyses. These PNG derived proteins were supplemented by domains from HB3, 3D7 and ItG parasites that included two DBLδ domains from group C PfEMP1s, a DC8 CIDRα1.1, a DC13 CIDRα1.4 and a CIDRα3.1 (Fig. 2b).

### Serology

The Luminex assay was performed as previously described [54]. For each protein an eleven point standard curve was made using two-fold dilutions of pooled positive plasma starting with 1/40, which was assigned an arbitrary value of 1000 relative units (RU). Plasmas were diluted in 0.02 % Tween-20, 0.1 % BSA in PBS pH 7.4. Fluorescent intensities of patients’ plasmas were used to interpolate antibody concentrations in RU from the standard curves.

ELISA was performed as previously described [54]. ELISA plates were blocked with 3 % (w/v) skim milk in PBS and all antibodies were diluted with 1 % (w/v) skim milk in PBS. Each plate included a pool of positive plasmas that was diluted two fold from 1/50 to 1/800 to generate a five point standard curve. The 1/50 dilution of pooled positive plasma was assigned an arbitrary value of 800 relative units (RU). All plasmas were tested at 1/50 and OD values interpolated from the standard curve for that plate. Any plasma that were below the curve were assigned the lowest value, any that were above the curve were re-tested at two-fold dilutions from 1/50 to 1/400. A pool of unexposed donor plasma at the same dilution as the test plasmas was included as a negative control in every Luminex and ELISA assay.

## Statistical analyses

The association between disease severity with age and parasitaemia was assessed using a Mann–Whitney *U*-test and with gender using a Fisher’s exact test. RU values for individual proteins were compared by Mann–Whitney *U*-test. To compare between patients with severe and uncomplicated malaria for antibody responses to proteins belonging to a single domain cassette or PfEMP1 group, patients were categorized according to whether their plasma sample lay above or below the median concentration of RU for that antigen: those above or equal scoring 1, or 0 if below. To derive a single quantitative score for each plasma for all the proteins belonging to a single DC or PfEMP1 group, the plasma’s scores for each antigen in that DC or group that were determined by either Luminex or ELISA were summed.

Any individual plasma with a score of 1 for any protein within a DC or PfEMP1 group was classified as a responder to that DC or PfEMP1. Individual plasma samples with a combined score of 0 for all proteins within a DC or group were classified as non-responders.

Differences in the proportions of severe and uncomplicated malaria patients whose plasma responded to a DC or PfEMP1 group were compared by contingency tables using Fisher’s exact test. Differences between severe and uncomplicated malaria patients in the number of proteins within a DC or PfEMP1 group to which patients responded were compared only for patients who responded to at least one protein in the DC or PfEMP1 group using Mann–Whitney *U*- tests. This indicated differences in the breadth of the response to PfEMP1s within that group. The patients that did not respond to any protein within the group, i.e. had a score of zero were not included to remove any biases associated with large frequencies of zero values in non-parametric comparisons [57].

## Results

### *Var* genes expressed in a Papua New Guinean cerebral malaria patient

To identify *var* sequences transcribed by parasites infecting this patient we used Illumina RNAseq to generate 65 bp paired-end short reads. Reads that mapped to the 399 full length *var* gene sequences available [4] were merged with reads that did not map to the *P. falciparum* 3D7 strain genome nor *Homo sapiens*, and the merged reads were subjected to two rounds of *de novo* assembly to generate 623 contigs that included 362 contigs with homology to *var* genes. The total length of assembled contigs was 200,158 bp, N50 423 bp, maximum contig length 4083 bp. The short contig length allowed assembly of only a few, full domains. However, the contigs could be aligned by BLASTN (E value <10^−5^) to individual domains from the 399 full-length *var* genes and orthologs of high identity to annotated domains and DC types were identified (Table 1).

**Fig. 1:**
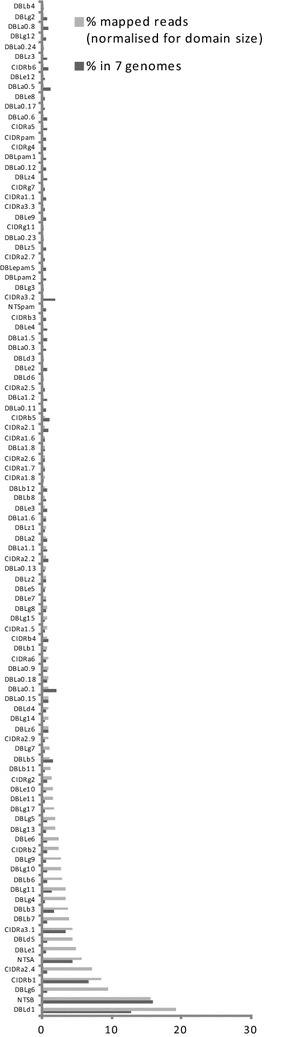
The sequence reads from the parasites infecting a cerebral malaria patient that assembled to different PfEMP1 domain subtypes normalized for domain size and expressed as a percentage of all reads that assembled to *var* contigs. Also shown is the percentage of total PfEMP1 domains that each domain subtype constituted in the seven sequenced *P. falciparum* genomes. Domains are ordered by transcript abundance

We compared the percentage of reads from the patient that assembled in transcripts with homology to domain subtypes with the percentage of total *var* exon 1 sequence in the seven sequenced *P. falciparum* genomes that each domain subtype represented. DBLδ1, NTSB, CIDRβ1, NTSA and CIDRα3.1 were all abundant transcripts but also constituted a similar proportion of *var* transcripts as the proportion of total *var* exon1 sequences they constitute in the seven sequenced genomes (Fig. 1). Therefore, their abundance could represent random *var* gene transcription. Other abundant transcripts (present at more than 10 % of the level of total DBLδ1) but which were transcribed at more than three times their level of representation in the seven sequenced genomes included DBLγ6, 4, 9, and 10, DBLδ5, DBLβ6 and 7, CIDRα2.4, CIDRb2 and DBLε1 and 6 (Fig. 1).

The abundance of individual transcripts was compared using reads mapped per kb of assembled contiguous transcript (RPK) (see “Methods” section). The most abundant individual transcripts included a diverse range of CIDR and DBL domains. The first and eighth most abundant transcripts were CIDRα2.4 and CIDRα3.1 sequences that could potentially bind CD36 [58] (Table 1). The second and third most abundant transcripts had no previous associations with severe disease or conserved domain cassettes. The fourth and the ninth most abundant were DC5 associated DBLδ5 sequences, 12 of the 13 published DBLδ5 sequences are present in DC5 genes. The 13th and 26th most abundant transcripts were also unique DC5 tandem-domain arrangements of DBLγ17-DBLδ5 and CIDRβ4-DBLβ3, respectively.

The most promising candidates for causing severe malaria are the EPCR-binding CIDRα1 domains of DC8 and DC13 PfEMP1s. The most abundant CIDRα1 capable of EPCR binding [36] in this patient was a CIDRα1.5-DBLβ7 contig ranked 50th overall by abundance. All described DC8s have a unique DBLα2-CIDRα1.1/1.6/1.8 tandem domain arrangement [4], but the most abundant DBLα2 was ranked 102nd by abundance. Four of the 50 most abundant transcripts had greatest homology to domains found in DC8 PfEMP1s (Table 1). Thus DC8 *var* genes may have been abundantly expressed by the parasites infecting this patient however these domains are also found at least as frequently in non-DC8 PfEMP1s. The DC13 is characterized by the tandem array of a DBLα1.7-CIDRα1.4 but neither of these domains were abundantly transcribed in this patient.

*Var1csa* sequences were the 7th, 12th and 45th most abundant transcripts; expression of this gene was not previously observed to be elevated in severe disease [59] and it is ubiquitously transcribed [59, 60], atypically late in the cell cycle after transcription of *var* genes encoding the adhesion phenotype [61, 62]. The 26th, 42nd and 35th most abundant transcripts were two DBLβ3 and a DBLβ5, respectively. DBLβ5 and some DBLβ3 including those in DC4 have been shown to bind ICAM-1 [63, 64]. DBLε10 from *var2csa* was the 43^rd^ most abundant transcript. No other DCs were identified in the 50 most abundant transcripts.

### Patterns of PfEMP1 antibody reactivity in severe and uncomplicated malaria in Papua

Antibody reactivity with PfEMP1 was assessed in plasma from 28 patients with severe malaria (median years 29, IQR 18.5-34; median *P. falciparum* parasites/µl 41,220, IQR 8260-334,273; 61 % male) and 35 patients with uncomplicated malaria (median years 22.5, IQR 18.0-25.5,; median *P. falciparum* parasites/µl 27,680, IQR 16,800-52,800; 54 % male). Patients with severe malaria tended to be older than those with uncomplicated malaria (*p* = 0.0599), but there was no significant difference in *P. falciparum* density (*p* = 0.7288) and gender (*p* = 0.7981). Twenty-three patients with severe malaria had a single diagnostic criterion (WHO) [48], including five with cerebral malaria, six with jaundice, eight with hyperparasitaemia, three with prostration, and one with acute renal failure. Five patients had two or more manifestations of severe malaria: one patient with jaundice and prostration, one with acute renal failure and acute respiratory distress syndrome, one with jaundice and hyperparasitaemia, and two with jaundice and acute renal failure.

**Fig. 2:**
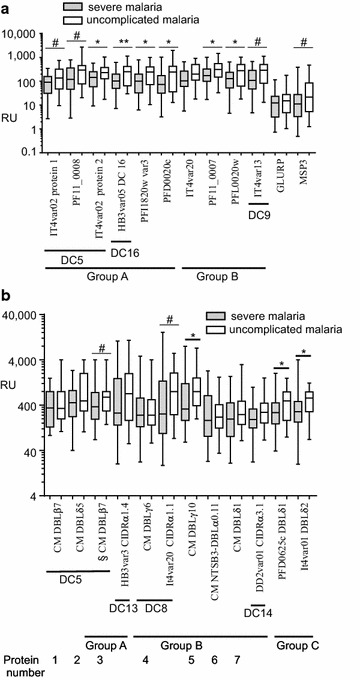
Levels of antibodies to PfEMP1s in plasma from 28 patients with severe malaria and 35 patients with uncomplicated malaria, RU (Relative units-see “Methods” section). Whiskers are minimum and maximum values, ^#^ *p* < 0.1, *p < 0.05, **p < 0.01. **a** Proteins assayed by Luminex; **b** Proteins assayed by ELISA; § insufficient of the DBLβ7 group A3 DC5 domain was available to test the full repertoire of plasma so it was only tested against 20 plasma from severe malaria patients, and was omitted from subsequent analyses

To examine the reactivity of Papuan patient plasma with different PfEMP1 groups and DCs previously associated with severe or uncomplicated disease we tested 10 PfEMP1 DCs and the non-PfEMP1 proteins GLURP and MSP3 by a Luminex multiplexed bead assay; and twelve recombinant, partial PfEMP1 domains by ELISA. The proteins tested included groupings previously associated with severe disease (DC8, DC13, DC5, group A and group B) (Fig. 2). Individual datapoints for all plasmas and all antigens tested are presented in Additional file 1.

The DC domain constructs used in the Luminex assay were derived from the 3D7, ItG and HB3 isolates. Seven of the proteins used for ELISA were derived from sequences transcribed in the PNG cerebral malaria patient described above and included abundantly transcribed (by RPK) representatives of DC5 DBLβ7, DC5 DBLδ5, DC8 DBLγ6 and DBLγ10 (Table 1; Fig. 2b and see “Methods” section for details). These domain subtypes were all transcribed at more than threefold their level of representation in the seven sequenced genomes (Fig. 1). The most abundantly transcribed representative of the conserved NTS-DBLα arrangement was also included as well as a less abundant DBLd1 transcript (protein 7) (Table 1; Fig. 2b). By Luminex assay, levels of antibodies to MSP3 but not GLURP were higher in plasma from patients with uncomplicated malaria than in severe malaria, (Fig. 2a) (median MSP3 22 versus 11 RU, respectively, *p* = 0.055). The MSP3 data suggests that patients with uncomplicated malaria may have had more prior exposure to *P. falciparum* infection than the patients with severe malaria.

In the Luminex assay, patients with uncomplicated malaria generally had higher levels of antibody to individual PfEMP1s than patients with severe disease, the greatest difference being in a DC16 PfEMP1 (p = 0.0095) (Fig. 2a). Interestingly DC16 are group A PfEMP1s that have been shown not to be associated with severe disease [38]. For four proteins, there was no significant difference (all *p* > 0.054) in antibody response between plasma from patients with severe and uncomplicated malaria. These proteins included three severe malaria-associated PfEMP1s, two DC5, and one DC8 (Fig. 2a).

In the ELISA, patients with uncomplicated malaria had significantly higher levels of antibody than patients with severe malaria to two group C PfEMP1s that were from lab isolates and a single group B DBLγ from the cerebral malaria patient (Fig. 2b; *p* < 0.05). A non-significant trend in the same direction (*p* < 0.10) was observed for a single DC5 and a single DC8.

**Fig. 3:**
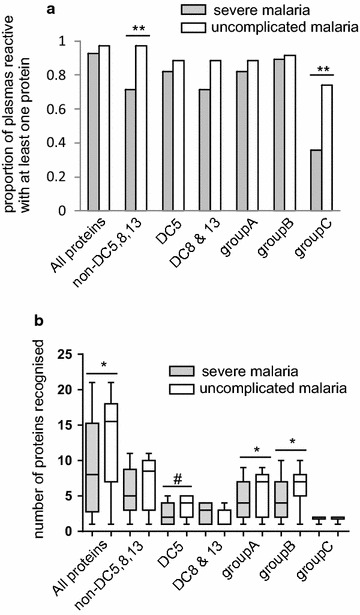
**a** The proportion of plasmas from patients with severe and uncomplicated malaria that had a response to a PfEMP1 group or DC (responders had greater than or equal to the median level of of antibody for all plasmas with at least one protein within the PfEMP1 group or DC-see “Methods section”) (Fisher’s exact test). **b** Amongst responders only, the number of proteins within a group or DC for which a response was detected, whiskers are minimum/maximum values, ^#^p < 0.1, *p < 0.05, **p < 0.01

The combined results for the ELISA and the Luminex assays revealed that a greater proportion of the patients with uncomplicated malaria than with severe malaria had antibodies to group C PfEMP1s (*p* = 0.004) and to PfEMP1s that were not DC5, nor 8, nor 13 (*p* = 0.008) (Fig. 3a). While the proportion of plasma reactive with severe malaria-associated proteins was higher in individuals with uncomplicated compared to severe malaria, the magnitude of the difference was smaller and not statistically significant (all *p* > 0.194). It is possible that analysing larger numbers of proteins in these groupings on a single platform may have detected differences.

Amongst patients with an antibody response to at least one protein of a cassette or group, plasma from patients with uncomplicated malaria recognized more PfEMP1s overall, and more group A and group B PfEMP1s than did plasma from patients with severe malaria (*p* = 0.031, *p* = 0.044 and *p* = 0.014, respectively, Fig. 3b). Thus although similar proportions of patients with severe and uncomplicated malaria had been exposed to at least one group A or group B PfEMP1 (Fig. 3a), the breadth of responses to the group A and group B PfEMP1s tested was lower in patients with severe malaria.

## Discussion

Sequencing the transcriptome of parasites causing CM in a single patient enabled assembly of a snapshot of the transcribed *var* repertoire in human malaria. Although entire genes could not be assembled, advances in the phylogenetics of *var* sequences [4] allowed the *var* contigs to be separated into useful classifications. Previous studies that implicated parasites expressing group A PfEMP1s [20, 23–25] and group B PfEMP1s [26] in severe disease provided no, or minimal, sequence data and were essentially restricted to classifying sequences to the groups defined by the *var* gene upstream sequences or by sequencing short DBLα tags.

The RNAseq of parasites causing CM in this PNG patient was consistent with previous studies of *var* genes in pathogenesis and abundantly expressed *var* genes identified included DC5 and possibly DC8, the DBLβ3 domain subtype and individual domains including CIDRα1.5 and DBLβ3. Recombinant proteins derived from the latter two domain subtypes have been shown to bind EPCR and ICAM-1 respectively [30, 36]. Other abundantly transcribed domain subtypes had not previously been identified in severe malaria. This limited study of a single patient indicates that RNAseq will be useful for identifying quantitative differences between transcribed *var* genes in severe disease in future studies.

The diversity of the transcribed *var* repertoire was consistent with a previous report of cerebral malaria in Africa [65]. However, 45 % of the reads that assembled into *var* transcripts were in the 20 most abundant *var* contigs that between them represented 27 domains. Thus the quantitative nature of RNAseq revealed a hierarchy of *var* transcript abundance in this patient’s peripheral blood that would be difficult to detect using the non-quantitative, nested RT-PCR approaches available to this previous study [65]. This suggests that the dominant *var* transcripts expressed by parasites causing cerebral malaria in a single patient are probably restricted in number.

Patients with uncomplicated malaria more commonly had antibodies to PfEMP1s that were from Group C or were not from DC5 nor DC8 nor DC13 than patients with severe malaria (Fig. 3a). In contrast, similar proportions of patients with severe and uncomplicated malaria had developed antibodies to the severe malaria associated PfEMP1s (group A and B, DC5, 8 and 13) (Fig. 3a), but the breadth of the response to group A and B PfEMP1s was greater in patients with uncomplicated than severe disease (Fig. 3b). Thus susceptibility to severe disease was associated with recognition of a narrower range of group A and B PfEMP1s and to an overall lack of antibodies to group C PfEMP1s and to PfEMP1s that were not DC5 nor DC8 nor DC13.

Overall, the serology findings in this Papuan adult population are consistent with existing models of infection in African children where parasites expressing severe malaria-associated group A and B PfEMP1s infect naive individuals and elicit antibodies [20, 66]. Susceptible, semi-immune individuals have antibody to some group A and B PfEMP1s [27], but protective immunity correlates with acquisition of antibodies recognizing a broader range of PfEMP1s [5–8]. Parasites expressing uncomplicated disease associated group C PfEMP1s, or PfEMP1s that were not DC5, 8 nor 13, would only dominate infections after parasites expressing severe disease associated PfEMP1s were controlled by acquisition of a broad antibody response. The alternative explanation is that group C PfEMP1s and PfEMP1s that were not DC5, 8 nor 13 were abundantly expressed by parasites causing acute, severe malaria but had not yet elicited antibodies. Although this cannot be excluded it is inconsistent with previous studies of *var* gene expression in both Africa and PNG [20–26].

## Conclusion

In Papuan adults severe malaria is associated with a lack of antibodies to non-DC5 and 8 and group C PfEMP1s in general, and with antibodies to a narrower repertoire of group A and group B PfEMP1s than in patients with uncomplicated malaria. These findings from Papua are consistent with reports from Africa of elevated group A and B *var* gene expression in severe disease [20, 23–26] and of earlier development of antibodies to group A PfEMP1s in children [27, 29]. This study has also established the feasibility of performing RNAseq on patient isolates to identify expressed *var* gene sequences.

## Additional file

10.1186/s12936-016-1296-4 Levels of antibodies to PfEMP1s in plasma from 28 patients with severe malaria and 35 patients with uncomplicated malaria, RU (Relative Units-see materials and methods). Median values are indicated by red bars and negative control pooled plasmas from unexposed donors by red dots, ^#^ *p* < 0.1, **p* < 0.05, ***p* < 0.01. A) Proteins assayed by Luminex; B) Proteins assayed by ELISA; § insufficient of the DBLβ7 group A3 DC5 domain was available to test the full repertoire of plasma so it was only tested against 20 plasma from severe malaria patients, and was omitted from subsequent analyses.
